# Fabrication of TiO_2_ Crystalline Coatings by Combining Ti-6Al-4V Anodic Oxidation and Heat Treatments

**DOI:** 10.1155/2015/395657

**Published:** 2015-02-15

**Authors:** María Laura Vera, Mario Roberto Rosenberger, Carlos Enrique Schvezov, Alicia Esther Ares

**Affiliations:** ^1^Instituto de Materiales de Misiones (IMAM), CONICET-UNaM, 1552 Félix de Azara Street, Posadas, 3300 Misiones, Argentina; ^2^Facultad de Ciencias Exactas, Químicas y Naturales, Universidad Nacional de Misiones, 1552 Félix de Azara Street, Posadas, 3300 Misiones, Argentina

## Abstract

The bio- and hemocompatibility of titanium alloys are due to the formation of a TiO_2_ layer. This natural oxide may have fissures which are detrimental to its properties. Anodic oxidation is used to obtain thicker films. By means of this technique, at low voltages oxidation, amorphous and low roughness coatings are obtained, while, above a certain voltage, crystalline and porous coatings are obtained. According to the literature, the crystalline phases of TiO_2_, anatase, and rutile would present greater biocompatibility than the amorphous phase. On the other hand, for hemocompatible applications, smooth and homogeneous surfaces are required. One way to obtain crystalline and homogeneous coatings is by heat treatments after anodic oxidation. The aim of this study is to evaluate the influence of heat treatments on the thickness, morphology, and crystalline structure of the TiO_2_ anodic coatings. The characterization was performed by optical and scanning electron microscopy, X-ray diffraction, and X-ray reflectometry. Coatings with different colors of interference were obtained. There were no significant changes in the surface morphology and roughness after heat treatment of 500°C. Heat treated coatings have different proportions of the crystalline phases, depending on the voltage of anodic oxidation and the temperature of the heat treatment.

## 1. Introduction

Titanium alloys are among the most commonly used materials in the manufacture of bone implants [1], cardiac valve parts [2, 3], stents, and pacemakers [4]. In general, the recognized properties of Ti alloys are mainly due to the formation, at room temperature, of a natural TiO_2_ oxide, which can reach a thickness of 2–10 nm. However, this native oxide can present poor superficial properties, such as low hardness and reduced resistance to wear and abrasion [5], so, it is necessary to deposit TiO_2_ coatings for the purpose of improving the properties. Among the different methods for obtaining TiO_2_ coatings, thermal and anodic oxidation are the simplest and the most economical [6]. The choice of the method depends on the requirements of the necessary surface depending on the tissue to which the prosthesis will be in contact. Rough and porous TiO_2_ coatings have good osseointegration [1], while for hemocompatible applications, smooth (average roughness less than 50 nm) and homogeneous surfaces are required [7].

The anatase and rutile are the most common crystalline phases of TiO_2_. The anatase is obtained at low temperatures in processes controlling the kinetics, while the rutile is formed in equilibrium conditions because it is the most stable thermodynamic phase. TiO_2_ amorphous phase usually crystallized in anatase phase at temperatures above 400°C, and the anatase transforms to rutile at temperatures around 600°C, depending on the preparation conditions, surface area, porosity, crystal size, and so forth [8].

According to other authors, the crystalline phases (anatase and rutile) are more biocompatible than the amorphous phase of TiO_2_, and some reports indicate that a mixture of anatase and rutile is the most suitable for hemocompatible applications [6, 9].

By the anodic oxidation technique, coatings of uniform thicknesses and low roughness which are amorphous are obtained at low voltages of oxidation. While above a certain voltage, which mainly depends on the electrolyte used, porous and crystalline coatings are formed [10]. In previous report we made a comparison between TiO_2_ coatings obtained by sol-gel and anodic oxidation processes [11] and found that anodic coatings copy the roughness of the substrate and the thickness is highly uniform.

The purpose of the present study is to obtain homogeneous and crystalline TiO_2_ coatings for hemocompatible applications by thermal treatments after the anodic oxidation of Ti-6Al-4V alloy. Thus, the influence of the anodic oxidation voltage and heat treatment temperatures was studied by analyzing the thickness, morphology, and crystalline structure of the coatings.

## 2. Experimental Methods and Materials

### 2.1. Surface Preparation

As a substrate to perform the oxidations, samples of Ti-6Al-4V (1 × 2) cm^2^ and 0.2 cm thick were used. These were roughened with SiC sandpaper with growing granulometry from number 120 to number 1500 and polished, first with diamond paste 1 *μ*m, lubricated with ethylene glycol, and then with a mixture 4 : 1 of colloidal silica (MasterMet, Buehler) and oxygenated water, to obtain a specular surface. Subsequently, samples were washed with detergent and water, rinsed with ethyl alcohol, and dried with hot air. The substrate sample was identified by TiG5.

### 2.2. Anodic Oxidation

The anodic oxidation was done at room temperature, circulating direct current between the anode of Ti-6Al-4V and a Pt wire cathode, spaced 5 cm from each other, immersed in a glass vessel containing a 1 M H_2_SO_4_ solution as electrolyte. The oxidation process was carried out at a constant voltage for 1 min. A different voltage per sample was used at 20 V, 40 V, and 60 V. Immediately after the oxidation, the samples were rinsed with deionized water and dried with hot air. These samples are identified with the value of the voltage at which they were oxidized, that is, 20, 40, and 60.

### 2.3. Heat Treatments

After anodizing, the heat treatments were carried out at two different temperatures 500 and 600°C. A heating rate of 10°C/min was used to achieve the desired temperature which was maintained for 1 h, using the muffle furnace. Furthermore, the same heat treatments (at 500 to 600°C) were performed to two substrates of Ti-6Al-4V (without anodic oxidation) to compare the effect of the thermal oxidation. Cooling was slowly done inside the furnace in all the cases.

Anodized and heat treated samples are named according to the voltage they were anodized followed by the heat treatment temperature, for example, 20-500. And the thermally oxidized samples are identified only with the heat treatment temperature, for example, 500.

The nomenclature used for each of the samples and the conditions in which they were obtained are presented in Table 1.

### 2.4. Characterization

The surface observation of the samples was performed by optical microscopy and scanning electron microscopy (SEM, FEI Quanta 200).

The thicknesses of the coatings were determined based on X-ray reflectometry measurements (XRR) using synchrotron radiation. This study was conducted in the D12A-XRD1 line at the National Laboratory of Synchrotron Light (LNLS, Campinas, Brazil) with a wavelength (*λ*) of 1.55015 Å.

XRR curves were plotted as intensity versus angle of incidence (*α*) and they are shown in Figure 2.

It can be seen that the intensity of the X-radiation from a maximum value decreases when increasing the angle of incidence but not in a monotonic mode, but it does so by presenting oscillations whose period is inversely related to the coatings thicknesses.

From the oscillations observed in XRR curves it was possible to calculate the thicknesses of the coatings using the modified Bragg's law [14]:

(1) $$\begin{array}{c} \alpha^{2}=\alpha_{c}^{2}+{\left(m+\Delta m\right)}^{2}\frac{\lambda^{2}}{4t^{2}}, \end{array}$$

where *α* corresponds to the maximum and minimum values of the incidence angles from the observed oscillations in Figure 2, *m* is the order of reflection, and Δ*m* takes values of Δ*m* = 0 for maximums or peaks and Δ*m* = 1/2 for the minimums of the curves. This is the case in which the film is less dense than the substrate [14]. *α*_*c*_ is the critical angle of incidence, below which the total reflection of X-rays was possible.

Measured values of *α* ^2^ versus (*m* + Δ*m*)^2^ were plotted for each sample and a linear correlation was calculated in each case, with *r* ^2^ values larger than 0.999, so that the slopes of each line are a function of the coatings thickness (*t*) according to (1). The calculated thickness values are shown in Table 1.

As shown in Figure 2, corresponding to the curve of sample 40-500, it does not sharp oscillations, so that the thickness was calculated using the discrete Fourier transform method [15]. The latter Fourier's method is based on the analysis of the power density of oscillating signals, so that it could be used to identify the main frequencies. Each of them is related to a unique layer thickness, and if a multilayer or fractions of layers are found on the sample, the XRR will be a linear combination of the frequency corresponding to each coating [15]. The same method was used to calculate the thickness of sample 60.

The relative roughness between the coatings and the substrate was qualitatively evaluated by analysing the different amplitudes of the oscillations of the XRR curves according to Parrat's theory [14, 16]. Moreover, the average roughness (*R*_*a*_) was measured using the Time Group TR200 equipment with a cut-off length of 0.8 mm, a sampling length of 0.8 mm, and a number of sampling length of 5.

The analysis of X-ray diffraction (XRD) to identify the oxide phases was performed using a Philips PW 3710 diffractometer with CuK*α* wavelength, using a thin-film Philips device that allows operation with a glancing incidence angle of 1°. This equipment was used for all the samples, except for the TiG5, 500, and 600 ones, whose diffraction patterns were obtained in the D12A-XRD1 line of the LNLS.

## 3. Results and Discussion

**Figure 1:**
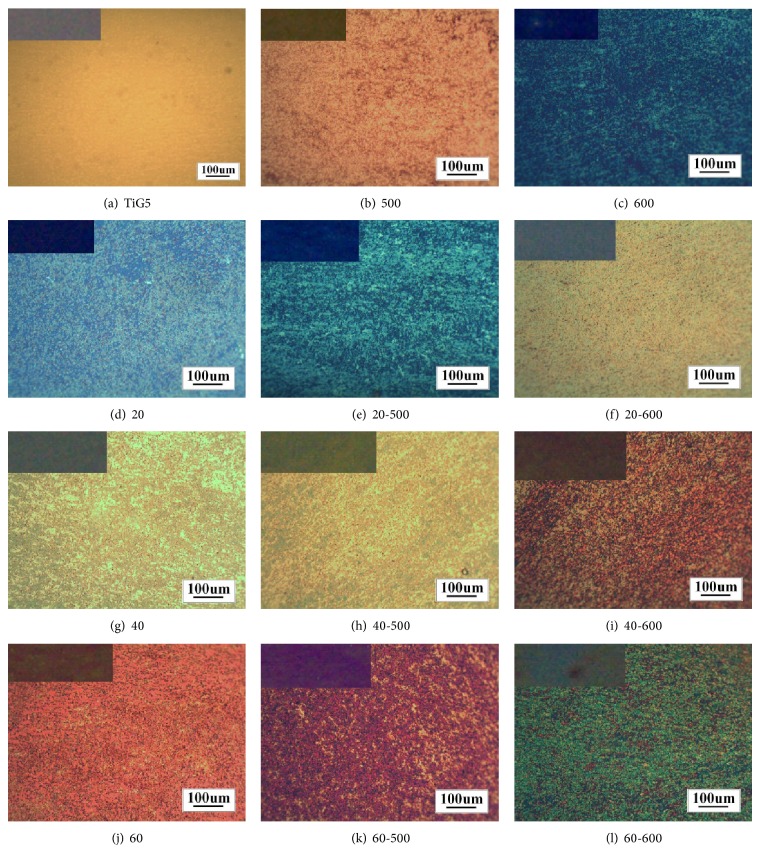
Macrographs and micrographs of anodically oxidized samples at 20, 40, and 60 V, with and without subsequent heat treatment at 500 and 600°C.

Figure 1 shows the different color coatings which were obtained depending on the applied voltage used in the anodic oxidation (first column of Figure 1) and the subsequent heat treatment temperature (second and third columns of Figure 1).

It may be noted that the colors of the samples without thermal treatment and the samples treated at 500°C were uniform, whereas the samples treated at 600°C show stains, probably because, at that temperature, the thermal oxidation which occurred was not uniform throughout the sample.

The optical micrographs of Figure 1 show that the samples, except the TiG5 one corresponding to the substrate, exhibit a dominant color with small portions of a different color, quasihomogeneously distributed over the surface. This pattern may be attributed to different crystallite orientations of the phases of the Ti-6Al-4V alloy used as substrate. The portions of the substrate with different crystalline orientations are associated with different oxide thicknesses and that would explain the different tone colors observed in the same samples. The difference in thickness according to the crystalline orientation was already observed in previous works [17, 18].

In both groups of micrographs it can be seen that, after the treatment at 500°C, the color intensified; however, after the treatment at 600°C a change in color was observed because of the thermal oxidation.

**Figure 2:**
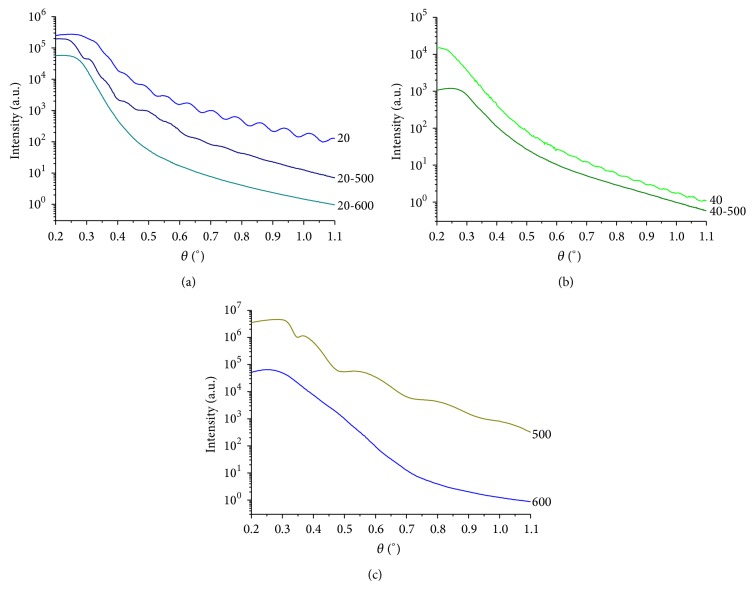
XRR curves of the samples oxidized by (a) anodic oxidation at 20 V with and without heat treatment; (b) anodic oxidation at 40 V with and without heat treatment; (c) thermal oxidation in air at 500°C and 600°C.

The colors of the samples were directly related to the thickness of the coatings [11, 19], which were calculated from XRR measurements (Figure 2) and are presented in Table 1.

The quantitative effect of heat treatment on the coatings thickness was analyzed based on the values of Table 1. Sample 20 has a thickness of 48 nm and sample 20-500 shows a thickness of 48 nm. Furthermore, sample 40 has a thickness of 92 nm, while the thickness of sample 40-500 is 100 nm, indicating that by the heat treatment at 500°C the thickness was increased by 9%. The little difference in the thickness of the coatings with and without heat treatment may explain the fact that they have similar colors. Both of them, although they seem to be similar, appear to have a slight difference, which could be because of a change in the optical properties of the films when the crystalline structure changes, due to heat treatment which will be discussed further in the following paragraphs.

Furthermore, sample 500 without anodic oxidation which was only heat treated at 500°C shows a coating with a thickness of 23 nm. After this result and considering that no greater increase was observed in the thickness of the anodized samples with the heat treatment at 500°C, it can be concluded that the anodic coatings act as barriers that prevent oxidation during heat treatment at 500°C.

The thicknesses of the heat treated samples at 600°C could not be calculated by XRR because fluctuations in the curves are virtually imperceptible as sample 20-600 curve shown in Figure 2(a). Some of the factors that may contribute to the smoothing of the oscillations are the coating thickness that increases the oscillation frequency or the change in coating optical density, due to crystallization by heat treatment, because the low contrast with the substrate reduces the amplitude of the oscillations. Diamanti et al. [20] also reported the absence of oscillations in reflectance curves corresponding to anodic coatings heat treated at 600°C.

The thickness of sample 600 from XRR curve could not be calculated (Figure 2(c)) because no oscillations were observed; however, it has been found in the literature that its thickness would be about 50 nm [12, 13]. This value is consistent with that calculated for sample 20 and in both cases the colors match (both of them are blue).

Regarding the effect of heat treatment performed on the anodic coatings at 600°C, although the thickness is not calculated by XRR, an increase in the thickness of the coating was evidenced by changes in color, which correspond, after the heat treatment, with the anodized samples to higher voltages than those used in each case. For example, the light green color of sample 20-600 is similar to sample 40 color, whose thickness is 92 nm (see Table 1). The effect is similar to sample 40-600 whose yellow and pink coloration corresponds to sample 60. Similarly, the sample 60-600 showed similar coloration to samples oxidized at 80 V [21]. de Las Heras [13] reported that with heat treatments at 600°C the increase in the thickness of the oxide was more evident than with those carried out at lower temperatures.

Analyzing the shape of the XRR curves in Figure 2, additional qualitative information was taken. According to the Parrat's theory of reflectometry in thin films [16], the variation in amplitude of the oscillations in the XRR scans in the monolayer films is related to the coating surface roughness (coating-air roughness) and the roughness of the coating-substrate interface. Since the substrates were prepared in the same way for every sample, they had the same substrate roughness and, therefore, the variations in the amplitude of the oscillations could be associated with the coating roughness.

In the case of samples 20, 40, and 500, the curves showed periodic oscillations indicating that they are uniform coatings of one layer. Moreover, the small amplitude of the oscillations indicates a low contrast between the densities of substrate and coating [15]. On the other hand, as the oscillations come to the end of the sweep with constant amplitude, it indicates that the roughness of the substrate and the oxide are the same order of magnitude, coinciding with the roughness values presented in Table 2.

Furthermore, the curve corresponding to sample 20-500 shown in Figure 2(a) has irregular oscillations, which could be because of a density gradient within the monolayer, which, as a result of heat treatment, from the viewpoint of reflection of X-rays, behaves as a multilayer [16]. Probably the same density gradient in the coating is responsible for the attenuation of the curves oscillations corresponding to sample 40-500 (Figure 2(b)) and sample 20-600 (Figure 2(a)) [16].

**Figure 3:**
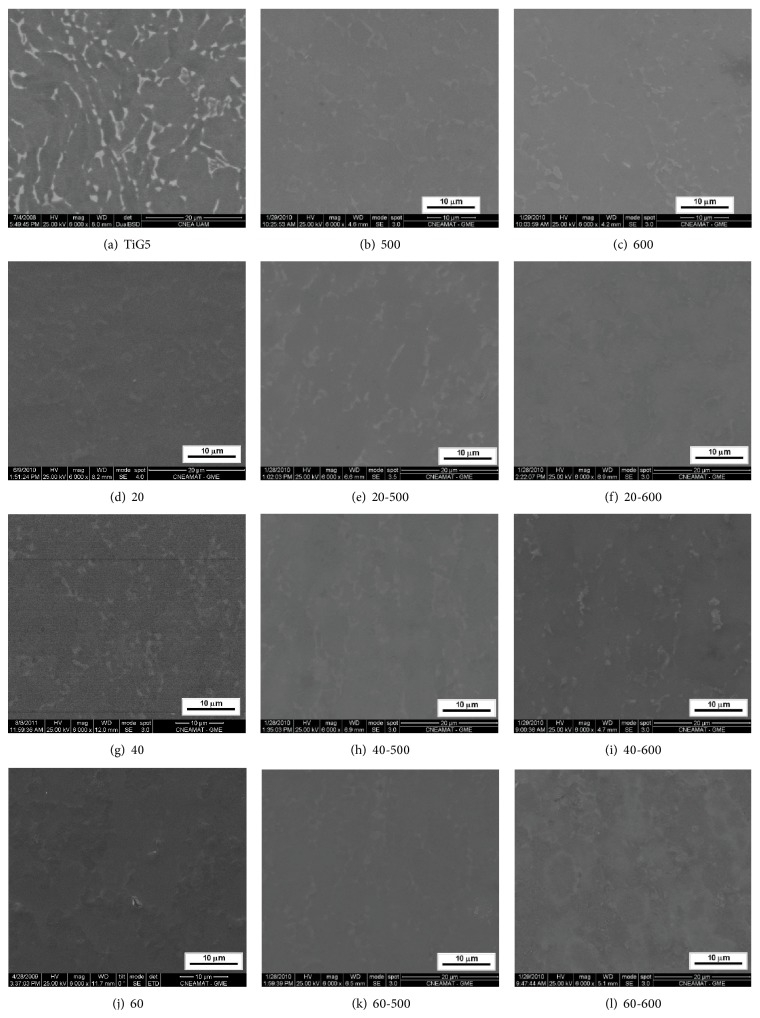
SEM micrographs of samples.

Regarding the morphology of the coatings, Figure 3 shows SEM micrographs of substrate (Figure 3(a)) and anodized and heat treated samples at 500°C and 600°C.

In Figure 3(a), corresponding to SEM micrograph of substrate, the *β* (bcc) phase in light gray and the *α* (hcp) phase of Ti-6Al-4V alloy in dark gray are observed.

In the SEM micrograph of Figures 3(b) and 3(c), in light grey, similar structures to *β* phase are observed indicating a selective oxide growth in the different phases of the substrate [22].

In the SEM micrographs of Figures 3(b) and 3(c) no significant difference between the surfaces of the oxides obtained at 500°C and 600°C for 1 h (samples 500 and 600) was observed. The evidenced morphology corresponds to a homogeneous surface layer. Kumar et al. [23] also observed homogeneity in the oxides grown at 500°C but at 24 h, which will indicate that the time at 500°C does not significantly affect the morphology of the oxides.

Regarding the anodic coatings without thermal treatment (Figures 3(d), 3(g), and 3(j)) in previous work it has been reported that by anodic oxidation of Ti-6Al-4V smooth and homogeneous coatings were obtained up to 60 V, and with greater profile and some pores from that voltage, due to the onset of the spark-discharge [10].

In the samples, heat treated at 500°C (Figures 3(e), 3(h), and 3(k)), a very little variation in the image was observed, which would indicate an area with low relief or with a very slight variation, so it can be concluded that the heat treatment at 500°C produces homogeneous and smooth surfaces and does not produce significant changes in the surface of the samples with respect to the samples which were only anodized.

In the case of sample 60, it was observed some pores. These pores were not observed after the treatment at 500°C (sample 60-500), Figure 3(k). This is in agreement with that reported by Masahashi et al. [24] with respect to the thermal treatments carried out at a similar temperature, 450°C, for anodic coatings promoted coalescence of the submicron pore producing smoother surfaces.

In the samples that were heat treated at 600°C (Figures 3(f), 3(i), and 3(l)), it is shown that the surface has a higher profile in all the cases that were analyzed, showing that it has a greater effect and it can be assumed that thermal oxidation may occur to some extent, even when the sample has an anodic coating.

Moreover, in SEM images of coatings obtained by anodizing, after subjecting them to heat treatment for 1 h at 600°C, several authors observed an increased amount of oxide on the surface, but no change of the morphology [25, 26].

Roughness values (*R*_*a*_) of the anodized samples with and without heat treatments are presented in Table 2. A slight increase in the values of *R*_*a*_ in the heat treated samples can be observed, giving an average of 0.03 *μ*m. This value is slightly higher than the samples which were only anodized (0.02 *μ*m). No significant difference between roughness values of the samples treated at different temperatures was observed.

**Figure 4:**
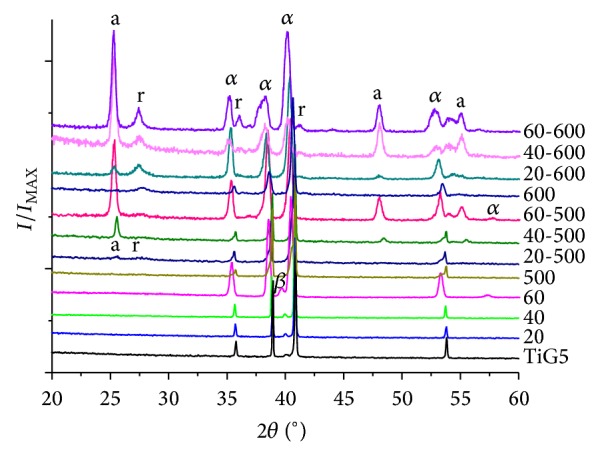
XRD patterns of the substrate and oxidized samples with different processes. a = anatase, r = rutile, *α* = *α* phase of TiG5, and *β* = *β* phase of TiG5.

The diffractograms of the samples obtained under glancing incidence of 1° are shown in Figure 4. As previously mentioned in the section of experimental procedure, the diffractograms of samples TiG5, 500, and 600 were obtained with different equipment as the other samples, and for this reason the peaks have a slightly different shape and they are displaced clockwise to the same phases. Therefore, these diffractograms were used for phase identification and not for detached determinations of deviations or changes in peak shape.

In Figure 4 it can be seen that the diffractograms of samples 20, 40, and 60, corresponding to anodic coatings (without heat treatment), and sample 500 do not show any anatase or rutile peak; only the corresponding peaks of the alpha and beta phases of Ti-6Al-4 V alloy were observed.

The absence of the peaks of oxide crystalline phases may be because the coatings were completely amorphous or crystalline fractions were too small to be detected by the technique used, since the coating thickness was very small and so was the mass. If we refer to the literature, there is controversy regarding the crystalline phases found in the anodic coatings. Some authors argue that the amorphous coatings grown at low voltages and from a threshold value close to 70 V begin to crystallize, first in the anatase phase and then in the rutile phase [27–30]. Other authors suggest that crystallization is gradual from low oxidation voltages; that is, the crystalline fraction increases with the voltage [12, 29, 31].

In Figure 4 it is also observed that all coatings anodized and heat treated at 500°C show peaks corresponding to the anatase and rutile phases. Anatase is a metastable phase, whose thermodynamic stability is higher than that of rutile when crystals are smaller than a critical value, which was reported between 11 nm and 45 nm [32]. The presence of rutile in the samples treated at 500°C may be related to the formation, on the metal-oxide amorphous interface, of the anatase crystallites exceeding that critical size, due to the surface effects of two-dimensional crystallization which transform to rutile following a nucleation and growth process [11]. This would favour the presence of rutile at lower temperature than 600°C proposed in the literature [8, 27]. As shown, the proportion of the rutile phase is not increased by the thickness of the initial anodic oxide. However, the intensity of the main anatase peak does increase with the anodizing voltage. This improvement can be explained considering that the voltage increases with increasing coating thickness and so does the mass of anatase.

Regarding the diffractograms of the samples that were heat treated at 600°C, it can only be observed that the coatings obtained by anodizing and heat treatment at 600°C showed peaks of anatase and rutile, wherein the anatase ratio also increases with anodization voltage. The presence of both phases observed in these samples would indicate that the anodic coating crystallized in anatase phase and likewise at 500°C, as previously mentioned, the anatase-rutile transition was started.

## 4. Conclusions

- Coatings of different colors depending on the applied voltage in the anodic oxidation and the subsequent heat treatment temperature were obtained. The anodic oxides have a barrier effect to treatment at 500°C and not with the 600°C. Besides the change of color, in some cases spots on coatings treated at 600°C were observed, indicating that this would be a very aggressive treatment.
- As regards the morphology of coatings, after heat treatment at 500°C, similar smooth surfaces to those of the anodic coatings were obtained at low voltages, even though they were more homogeneous than those presenting pores. However, with treatment at 600°C, there was evidence of increased texture by thermal oxidation.
- As regards the roughness, a slight increase in the values of *R*_*a*_ in the samples with heat treatment (at both temperatures) was noted, giving an average of 0.03 *μ*m, slightly higher than the value of the samples which were only anodized (0.02 *μ*m), but they were still within the desirable limits for application as coatings of heart valve (*R*_*a*_ < 0.05 *μ*m).
- As regards the thickness, there was an average increase of about 9% with heat treatment at 500°C which did not result in a color change of the coating. However, the increase was more noticeable with the treatment at 600°C as a change in color corresponding to 20 V that was observed in voltage-color-thickness scale established for anodizing with 1 M sulphuric acid without heat treatment.
- All coatings were heat treated resulting crystalline, with the presence of the anatase and rutile phases in different ratios, depending primarily on the thickness of anodic oxide growth at different voltages and the temperature of the heat treatment.
- The presence of crystalline phases modified XRR curves, with respect to monolayers samples which were only anodic or thermally oxidized, indicating that they correspond to a multilayer structure with different densities.

For hemocompatible applications, the optimum processes conditions are those which give crystalline TiO_2_ coating of homogeneous thickness and low roughness (*R*_*a*_ < 0.05 *μ*m). It has been found that the anodic oxidation process at low voltages without thermal treatment produced a homogeneous, smooth surface but it did not assemble the combination of crystalline phases sought. On the other hand, the processes of anodic oxidation at high voltages (postspark) and the heat treatment at 600°C produce crystalline samples, which had rough surfaces and pores.

Among the results of tests presented in this research, the conditions that gave best results were anodic oxidation realized at 20 V to 60 V, with thicknesses of 48 nm to 130 nm, followed by heat treatment of 1 h at 500°C, which does not affect the thickness and morphology of coatings.

## Figures and Tables

**Table 1:** Conditions under which the different samples, thicknesses, and detected crystalline phases were obtained.

Sample	Color	Anodizing voltage [V]	Conditions of thermal treatment (time [h] and temperature [°C])	Thickness [nm]	Crystalline phase of TiO_2_^d^
20	Blue	20	—	48^a^	—
40	Green	40	—	92^a^	—
60	Pink and yellow	60	—	130^b^	—
500	Golden	—	1 h, 500°C	23^a^	—
20-500	Blue	20	1 h, 500°C	48^a^	a + r
40-500	Green	40	1 h, 500°C	100^a^	a + r
60-500	Pink and yellow	60	1 h, 500°C	130^b^	a + r
600	Blue	—	1 h, 600°C	50^c^	r
20-600	Green	20	1 h, 600°C	92^b^	a + r
40-600	Pink and yellow	40	1 h, 600°C	130^b^	a + r
60-600	Dark green and pink	60	1 h, 600°C	192^b^	a + r

^a^Values measured by XRR; ^b^calculated using measured values and oxidation conditions; ^c^estimated value using [12, 13]; ^d^a: anatase, r: rutile.

**Table 2:** Roughness values, *R*_*a*_, of the samples.

Sample	*R*_*a*_ [*μ*m]
TiG5	0.020
20	0.019
40	0.020
60	0.018
500	0.021
20-500	0.017
40-500	0.025
60-500	0.030
600	0.029
20-600	0.030
40-600	0.030
60-600	0.040
